# Malignant Fibrous Histiocytoma of The Pancreas

**DOI:** 10.1155/1989/60313

**Published:** 1989

**Authors:** J. F. W. Garvey, A. Ng, J. F. England, D. M. Sheldon

**Affiliations:** 1 The Department of Surgery and Department of Anatomical Pathology Royal Prince Alfred Hospital Missenden Road Camperdown Sydney Australia; 2 R.P.A.H. Medical Centre 100 Carillon Avenue NEWTOWN Sydney NSW 2042 Australia; 3 The Canterbury Hospital Australia

## Abstract

A case of fibrous histiocytoma of low grade malignancy arising from the uncinate lobe of the pancreas is reported. This is an unusual site for these extremely rare tumours. Survival up to 4 years has been achieved in our patient following surgical resection.


*HPB Surgery* 1989, Vol. 1, pp. 233–237
Reprints available directly from the publisher
Photocopying permitted by license only

© 1989 Harwood Academic Publishers GmbH
Printed in Great Britain

### CASE REPORT
### MALIGNANT FIBROUS HISTIOCYTOMA OF THE PANCREAS

J.F.W. GARVEY[1], A. NG,[2] J.F. ENGLAND[3] and D.M. SHELDON[4]*

*The Department of Surgery and Department of Anatomical Pathology, Royal Prince Alfred Hospital, Missenden Road, Camperdown, SYDNEY*

*(Received 11 August 1988)*

A case of fibrous histiocytoma of low grade malignancy arising from the uncinate lobe of the pancreas is reported. This is an unusual site for these extremely rare tumours. Survival up to 4 years has been achieved in our patient following surgical resection.

KEY WORDS: Adult, pancreas neoplasms, fibrous histiocytoma, pathology, treatment, case report.

### INTRODUCTION

Malignant fibrous histiocytoma is a rare soft tissue sarcoma of variable behaviour that is usually found in the subcutaneous tissue of the upper and lower limbs[1]. Less common primary sites are the retroperitoneal tissues and the head and neck. This report describes a patient with a fibrous histiocytoma of low grade malignancy arising from the uncinate lobe of the pancreas where early presentation and surgical treatment have resulted in a curative resection.

### CASE REPORT

A 77 year old male presented in February 1983 with anorexia, epigastric discomfort, heartburn, weight loss of 16 kg in nine months and general malaise. On examination, he was thin, with a blood pressure of 180/80 mm Hg and a pulse rate of 70/min. There was a non-tender mass approximately 10 cm in diameter palpable in the epigastrium and this was not attached to superficial structures and did not move with respiration. CAT scan of the abdomen confirmed the presence of a large rounded mass

* Correspondence to: Mr. D.M. Sheldon, R.P.A.H. Medical Centre, 100 Carillon Avenue, NEWTOWN NSW 2042.
[1]Senior Surgical Registrar, The Canterbury Hospital,[2] Head, Department of Anatomical Pathology, Royal Prince Alfred Hospital,[3] Physician, Royal Prince Alfred Hospital,[4] Visiting Surgeon, Royal Prince Alfred Hospital approximately 10 cm × 8 cm in the region of the head of the pancreas. There was no para-aortic lymph node enlargement. Coeliac axis angiography indicated that the mass was supplied by the gastroduodenal artery and vessels arising from the head of the pancreas. Biochemical screen was normal but the serum amylase estimation was at the upper limit of normal (85 units per litre).

*Operation Report*

On 9th February 1984, the abdomen was explored through a right upper paramedian laparotomy incision. A mobile lobulated tumour was found arising from the inferior aspect of the pancreas immediately to the right of the superior mesenteric vein. The tumour had a well defined capsule and could be readily dissected away from the adjacent duodenum and transverse colon. Care was required to avoid injury to the superior mesenteric vessels on the medial side of the tumour. The base of the tumour was attached to the inferior aspect of the pancreas in front of the third part of the duodenum. The penduculated tumour mass was removed with a narrow cuff of pancreatic tissue. A short vascular pedicle arose from the inferior pancreatico-duodenal vessels. The pedicle was secured and the abdomen closed after inserting a suction drain.

*Pathology*

Macroscopically, the resected tumour was a lobulated, rubbery mass of tissue measuring 12.5cm × 10cm × 6cm and weighed 337 grams (Figure 1). The cut surface was tan in colour and there was a small central area of calcification. Microscopically, the tumour was composed of whorls of spindle cells with bundles of collagen tissue (Figure 2). Foci of polygonal cells with foamy cytoplasm indicating a histocytic component were evident (Figure 3). Mitotic figures averaged 2 per 10 high power field. The tumour contained a fine thin capsule. These findings were compatible with a diagnosis of fibrous histiocytoma of low grade malignancy. Electron microscopy examination showed that the tumour was of fibrohistiocytic type widely scattered within a matrix containing numerous collagen bundles, fibrillar material and considerable numbers of plasma cells. The majority of the cells were of the fibroblastic type with isolated histiocytic cells.

### DISCUSSION

Malignant fibrous histiocytoma of the gastrointestinal tract is being reported more frequently[2]. One probable case of malignant fibrous histiocytoma of the pancreas has been reported[3] in a patient who presented with obstructive jaundice. On that occasion, the tumour arose in the head of the pancreas and necessitated pancreatico-duodenectomy.

In the Japanese literature a case of malignant fibrous histiocytoma of the pancreas arising from the tail of the pancreas in a 44 year old male has been reported[4]. This patient presented with fever, weight loss and fatigue and there was a mass measuring 25cm in diameter in the left upper quadrant of the abdomen. The tumour was imaged

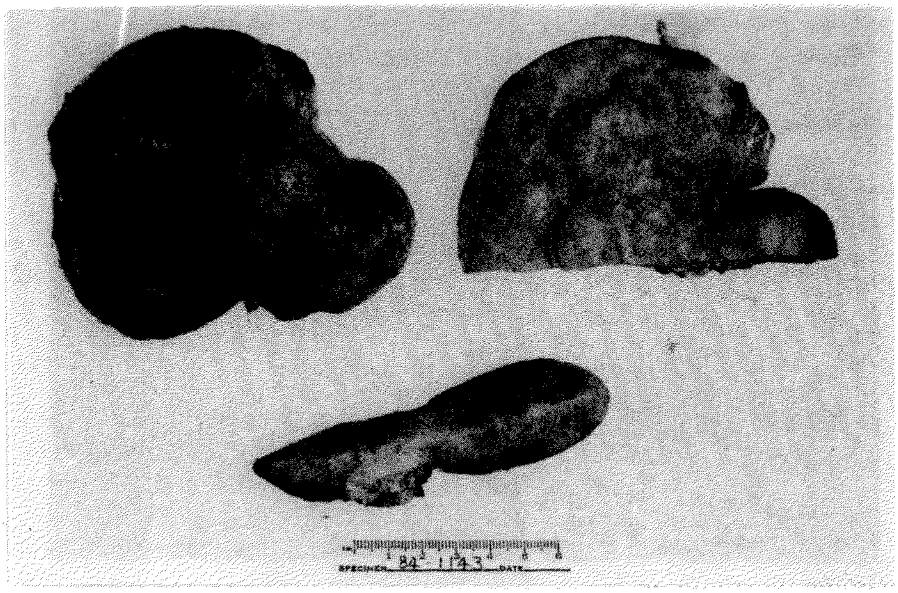

**Figure 1** External lobulated appearance of tumour (top left). Cut surface of tumour showing nodularity separated by cleft-like spaces (top right). Remainder of cut surface showing portion contiguous with uncinate lobe of pancreas (bottom).

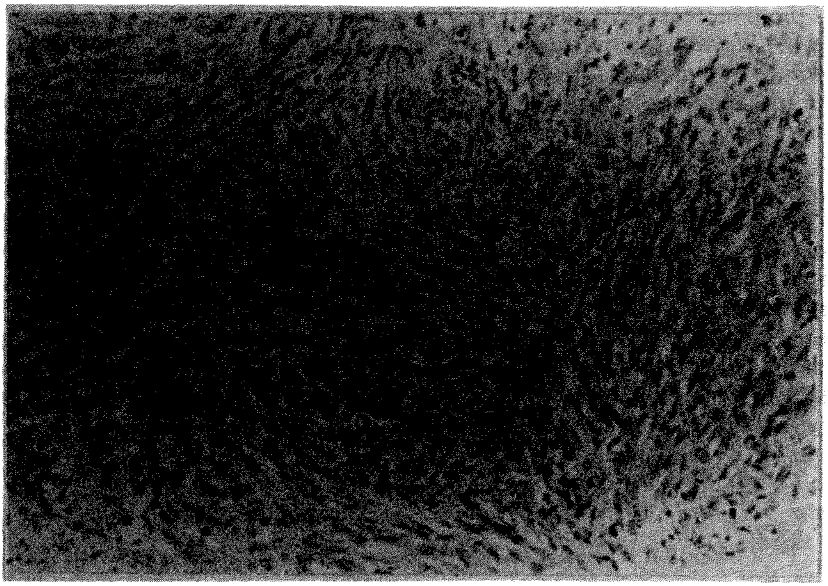

**Figure 2** Whorling pattern of spindle cells of fibroblastic origin H+E 60 X.

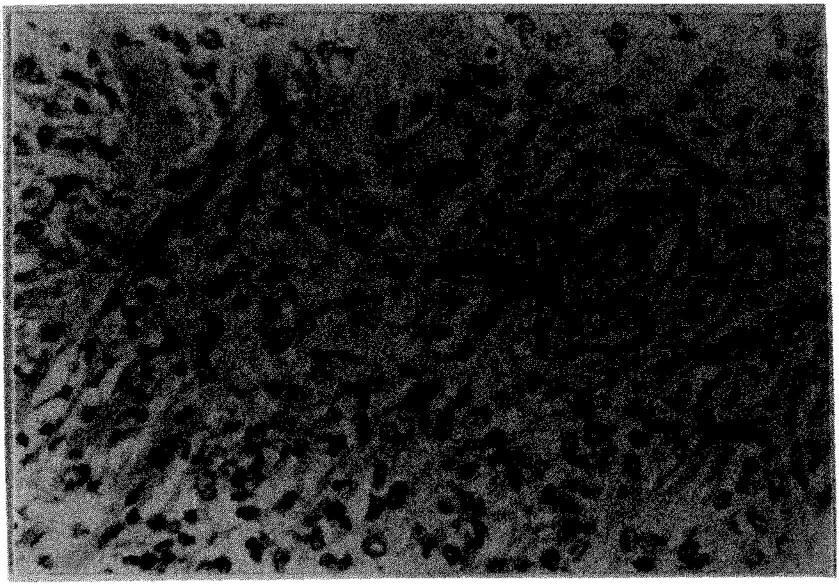

**Figure 3** Polyglonal cells with foamy cytoplasm of histiocytic origin intermingled with spindle fibroblastic cells. H+E 200 X.

by CAT scan, ultrasonography and $^{67}$Ga-citrate scintigraphy. Coeliac axis angiography showed it to be supplied by branches of the pancreatic artery from the body of the pancreas. This patient was operated on the 5/3/85 and a tumour which weighed 8.16kg was removed (operative details not documented). This patient was evidently alive 15 months after the operation.

In our patient, the proximity of the tumour to the duodenum resulted in a comparatively early clinical presentation due to obstructive symptoms and, at laparotomy, the tumour was easily dissected from the surrounding duodenum and superior mesenteric vessels.

The role of adjuvant chemotherapy and radiotherapy for this type of tumour has been canvassed but the response to treatment has been variable. Certainly when metastases are present these treatment modalities afford little prolongation of the median survival time[5].

Ultimate prognosis for a patient from whom this type of tumour has been removed is difficult to estimate due to the paucity of data, but surgical resection of the tumour was possible in our patient and there were no demonstrable lymph nodes involved. He remains alive and well 4 years following resection.

### *References*

1. Pillay, S.P., Roberts, S.J., Thynne, G.S.J., and Hardie, I.R. (1980). Malignant fibrous histiocytoma, Australia, *New Zealand Surg;* **50**, 532–7.
2. Sewell, R., and Levine, B.A., Harrison, G.K., Tio, F., and Schwesinger, W.H., (1980) Primary malignant fibrous histiocytoma of the intestine: Intussusception of a rare neoplasm. *Dis. Col. and Rect;* **23**, 198–201.

3. Margules, R.M., Allen, R.E., Dunphy, J.E., (1976) Pancreatic tumour of mesenchymal origin presenting as obstructive jaundice. *Am. J. Surg.,* **131**, 357–9.
4. Ishiguchi, T., Shimamoto, K., Kaii, O., Sakai, M., Ishigaki, T., Sakuma, S., Hasegawa, H. and Nimura, Y. (1986). Malignant fibrous histiocytoma of the pancreas. *Rinsho Hoshasen;* **31**, 655–8.
5. Yeh, H.C., and Wolf, B.S. (1977) Ultrasonography and computed tomography in the diagnosis of homogenous masses. *Radiology;* **123**, 425–8.

